# Prevention of postpartum haemorrhage: cost consequences analysis of misoprostol in low-resource settings

**DOI:** 10.1186/s12884-015-0749-z

**Published:** 2015-11-23

**Authors:** Danielle L. Lang, Fei-Li Zhao, Jane Robertson

**Affiliations:** Clinical Pharmacology, School of Medicine and Public Health, Faculty of Health and Medicine, University of Newcastle, Callaghan, NSW Australia

**Keywords:** Misoprostol, Postpartum haemorrhage, Cost-consequences analysis

## Abstract

**Background:**

While inferior to oxytocin injection in both efficacy and safety, orally administered misoprostol has been included in the World Health Organization Model List of Essential Medicines for use in the prevention of postpartum haemorrhage (PPH) in low-resource settings. This study evaluates the costs and health outcomes of use of oral misoprostol to prevent PPH in settings where injectable uterotonics are not available.

**Methods:**

A cost-consequences analysis was conducted from the international health system perspective, using data from a recent Cochrane systematic review and WHO’s Mother-Baby Package Costing Spreadsheet in a hypothetical cohort of 1000 births in a mixed hospital (40 % births)/community setting (60 % births). Costs were estimated based on 2012 US dollars.

**Results:**

Using oxytocin in the hospital setting and misoprostol in the community setting in a cohort of 1000 births, instead of oxytocin (hospital setting) and no treatment (community setting), 22 cases of PPH could be prevented. Six fewer women would require additional uterotonics and four fewer women a blood transfusion. An additional 130 women would experience shivering and an extra 42 women fever. Oxytocin/misoprostol was found to be cost saving (US$320) compared to oxytocin/no treatment.

If misoprostol is used in both the hospital and community setting compared with no treatment (i.e. oxytocin not available in the hospital setting), 37 cases of PPH could be prevented; ten fewer women would require additional uterotonics; and six fewer women a blood transfusion. An additional 217 women would experience shivering and 70 fever. The cost savings would be US$533.

Sensitivity analyses indicate that the results are sensitive to the incidence of PPH-related outcomes, drug costs and the proportion of hospital births.

**Conclusions:**

Our findings confirm that, even though misoprostol is not the optimum choice in the prevention of PPH, misoprostol could be an effective and cost-saving choice where oxytocin is not or cannot be used due to a lack of skilled birth attendants, inadequate transport and storage facilities or where a quality assured oxytocin product is not available. These benefits need to be weighed against the large number of additional side effects such as shivering and fever, which have been described as tolerable and of short duration.

## Background

Postpartum haemorrhage (PPH) continues to be a leading cause of maternal deaths, particularly in low-income countries [1, 2]. Almost one-third of maternal deaths worldwide are due to haemorrhage, mostly in the postpartum period [3]. It is therefore recommended that active management of the third stage of labour (AMTSL) be offered to all women during childbirth by a skilled attendant to prevent PPH [4]. WHO guidelines for AMTSL include prophylactic administration of a uterotonic soon after the birth of the baby, delivery of the placenta by controlled cord traction (where skilled birth attendants are available) and late cord clamping (performed after 1 to 3 min after birth) [4]. The latter is not recommended in all guidelines [5]. Even with these efforts to prevent PPH, some women will require treatment for excessive bleeding and timely interventions including use of additional uterotonics by skilled providers [6].

Drugs that can be used for PPH prophylaxis include oxytocin (intravenous (IV) or intramuscular (IM)); syntometrine (IM); ergometrine (IV or IM) and oral misoprostol [7]. The gold standard treatment for the prevention of PPH is 10 iU oxytocin, recommended by the World Health Organization (WHO), the International Federation of Gynecology and Obstetrics and International Confederation of Midwives [4, 5]. However, widespread use of oxytocin is impeded by its need for cold-chain storage and either intravenous or intramuscular administration by a skilled birth attendant. In the world’s least developed countries it is estimated that only 35 % of births are attended by skilled health workers [8]. While new aerosol formulations of oxytocin are being developed that do not require skilled health workers for administration or cold chain storage, these products remain in development. In addition, problems with the quality of the oxytocin product available have been identified in some settings [9]. A recent study by the Food and Drugs Authority Ghana highlighted problems with the quality of oxytocin in that country, finding that the majority of samples of oxytocin (65.5 %) did not meet the required standards for quality [9].

Misoprostol is a tablet alternative and has the advantages of being stable at room temperature, easy to administer, and widely accessible [10], features especially critical in lower resource settings [8]. In early 2011 misoprostol was added to the World Health Organization’s Model List of Essential Medicines for the prevention of PPH as a safe and effective medicine [11].

A recent review article questioned WHO’s decision to include misoprostol on the WHO Model List of Essential Medicines on the basis of a lack of evidence of efficacy [12]. The authors of the review subsequently submitted an application to WHO’s 19th Expert Committee on the Selection and Use of Essential Medicines in Geneva (April 2013) to have misoprostol for PPH prevention deleted from the Model List of Essential Medicines on the basis of the poor quality of the evidence [13]. While there is some heterogeneity and potential for bias in the published misoprostol trials, there is evidence of a reduction in PPH when compared with placebo [14]. The decision of the Expert Committee was to retain misoprostol on the Essential Medicines List [15].

A recent Cochrane systematic review, Tunçalp [14], of prostaglandins for preventing postpartum haemorrhage has compared the efficacy and safety of misoprostol versus placebo and misoprostol versus oxytocin. Compared with ten iU injectable oxytocin, 600 μg oral misoprostol was associated with higher risks of PPH, severe PPH and use of additional uterotonics. There was a trend towards a reduced risk of blood transfusion with misoprostol, but this did not reach statistical significance. Compared with placebo, 600 μg oral misoprostol was associated with reduced risks of PPH and blood transfusion. There was no statistically significant difference between treatments in severe PPH events. More events of shivering and fever were reported with misoprostol compared with both oxytocin and placebo. The trials included in the Tunçalp 2012 review [14] were conducted in both hospital and community settings, under which reported results might vary.

Differences in the rates of PPH and other outcomes of interest in various settings can lead to different conclusions regarding the cost-effectiveness of a program of PPH prevention using misoprostol or oxytocin. The pattern of health resource utilisation associated with a strategy of prevention of PPH differs in the community compared to hospital settings as well. Existing economic evaluations for misoprostol in PPH prevention have focussed on community facilities only [16, 17]. We therefore carried out an economic analysis of misoprostol use for PPH prophylaxis in different settings, drawing on data from the 2012 Cochrane systematic review [14].

## Methods

A cost-consequences analysis was conducted from the international health system perspective, which presents the incremental costs, incremental effectiveness and incremental safety associated with misoprostol versus its comparators, without calculating an incremental cost-effectiveness ratio. This allows each of the outcomes associated with the prevention of PPH to be considered without converting outcomes to a single measure, such as quality adjusted life years; allowing decision makers to make their own trade-offs between outcomes [18].

Several options were examined. First, that only oxytocin is available and used in the hospital or health centre where skilled birth attendants are trained in the administration of intravenous (IV) or intramuscular (IM) oxytocin and appropriate storage and transport facilities are available. In this scenario, no preventive treatment for PPH is offered in the community setting. In the second scenario, oxytocin is used in the hospital or health centre and misoprostol is used in the community setting. In the third setting, misoprostol is used in both hospital and community settings. The latter might be considered where oxytocin is not available or the quality of the product supplied cannot be assured.

Two comparisons are presented in the cost consequences analysis - the first assumed that oxytocin would be used in the hospital setting and misoprostol would be used in the community setting. For this analysis the comparator is oxytocin used in the hospital setting and no treatment offered in the community. The second analysis assumed that misoprostol would be used in both hospital and community settings. For this comparison, the comparator is no treatment in either hospital or community settings.

For both analyses, the base case assumed that 40 % of births occur in the hospital/health centre setting and 60 % of births occurred in the community setting. These proportions were varied in sensitivity analyses.

The primary outcome of interest was PPH events, defined as blood loss ≥500 mL. Severe PPH was defined as blood loss ≥1000 mL. Use of additional uterotonics, need for blood transfusion, shivering and fever were also included in the analyses. To simplify the analyses, and due to a lack of evidence of a difference between treatments, several outcomes (and their associated costs) were not included in the analyses: maternal death, hysterectomy, manual removal of placenta, nausea, vomiting and diarrhoea. The baseline event rates without treatment and with oxytocin treatment were obtained from the most recent Cochrane systematic review, Tunçalp [14] (placebo rates from the misoprostol 600 μg versus placebo comparison; oxytocin rates from the misoprostol 600 μg versus injectable uterotonics comparison) (Table 1), as were the relative risks of misoprostol compared with placebo and oxytocin (Table 2). Where relative risks (and their 95 % confidence intervals) were not generated in Tunçalp [14] due to heterogeneity, these were generated for the analysis using the random effects model in StatsDirect (Version 2.7.8, 2010), so that incremental outcomes and costs could be calculated. Numbers of events were based on a cohort size of 1000 women and derived by multiplying the baseline event rates for each outcome by the relative risks associated with each treatment option.

**Table 1:** Baseline event rates used in the economic evaluation

Baseline incidence	Point estimate	Low	High
Placebo event rates (Tunçalp [14])			
PPH	16.0 %	12.0 %	27.3 %
Severe PPH	3.0 %	0.7 %	9.7 %
Additional uterotonics	7.2 %	0.7 %	38.7 %
Blood transfusion	0.8 %	0.5 %	0.9 %
Shivering	10.8 %	0.0 %	17.3 %
Fever	1.6 %	0.0 %	4.3 %
Oxytocin event rates (Tunçalp [14])			
PPH	12.4 %	0.4 %	17.3 %
Severe PPH	2.7 %	0.0 %	6.5 %
Additional uterotonics	11.1 %	4.4 %	14.0 %
Blood transfusion	1.1 %	0.0 %	1.6 %
Shivering	6.0 %	0.0 %	40.4 %
Fever	1.0 %	0.00 %	7.00 %
Placebo event rates; alternative values: home birth setting (Hundley [23])			
PPH	12.2 %	NC	NC
Severe PPH	1.2 %	NC	NC
Additional uterotonics	5.4 %	NC	NC
Blood transfusion	2.9 %	NC	NC
Shivering	17.8 %	NC	NC
Fever	13.8 %	NC	NC

*Abbreviations*: *NC* not calculated. Placebo event rates from Tunçalp [14] based on the misoprostol 600 μg vs placebo comparison; oxytocin event rates from the misoprostol 600 μg vs injectable uterotonics comparison. Point estimates derived from an average across all trials; Low values derived from the trial with the lowest estimate; High values derived from the trial with the highest estimate

**Table 2:** Relative risks used in the model

Comparison	Point estimate	Low	High
RR misoprostol vs oxytocin (Tunçalp [14])			
PPH	1.43	1.34	1.52
Severe PPH	1.36	1.17	1.58
Additional uterotonics	1.35	1.1	1.66
Blood transfusion	0.77	0.59	1.02
Shivering	2.94	2.35	3.67
Fever	6.77	5.55	8.27
RR misoprostol vs placebo (Tunçalp [14])			
PPH	0.77	0.6	0.99
Severe PPH	0.91	0.51	1.63
Additional uterotonics	0.86	0.66	1.13
Blood transfusion	0.24	0.06	0.94
Shivering	3.01	2.68	3.39
Fever	5.39	3.78	7.69
RR misoprostol vs placebo alternative values (Hundley [23])			
PPH	0.58	0.38	0.87
Severe PPH	0.2	0.04	0.91
Additional uterotonics	0.34	0.16	0.73
Blood transfusion	0.16	0.07	0.38
Shivering	2.18	1	4.72
Fever	1.4	0.16	12.09

*Abbreviations*: *RR* relative risk, *PPH* postpartum haemorrhage. Low values are the lower 95 % confidence limit; High values are the upper 95 % confidence limit from the relevant review

Resource use associated with PPH treatment was based on WHO’s Mother-Baby Package Costing Spreadsheet [19]. The Spreadsheet and accompanying Users’ Manual developed by WHO is a tool for estimating the cost of implementing a set of interventions at the district level to reduce maternal and newborn mortality and morbidity. The clinical management of PPH summarised from the Spreadsheet Users’ Manual is presented in Table 3. According to the WHO Mother-Baby Package, the treatment of PPH consists of IM/IV oxytocin immediately after delivery, uterine massage, catheterisation of the bladder if necessary, repair of tears, fluids and blood transfusion, and referral as necessary. Health posts (staffed by auxiliary workers) should refer all cases to a higher level in the health system following initiation of treatment. Health centres (staffed by at least one doctor, professional nurse or midwife) are assumed to refer approximately half of their cases to the hospital.

**Table 3:** Clinical management of postpartum haemorrhage

Component	Health post	Health centre	Hospital
Treatment	-	-IM/IV oxytocin immediately after delivery;	-IM/IV oxytocin immediately after delivery;
		-uterine massage;	-uterine massage;
		-catheterisation of bladder if necessary;	-catheterisation of bladder if necessary;
		-repair of tears; and	-repair of tears;
		-fluids	-fluids and
			-blood transfusion
No. hospital days	-	50 % will require 2 days	50 % will require 7 days
Referral	100 %: 50 % to health centre; 50 % to hospital	50 % to hospital	-

Source: WHO Mother-Baby Package Costing Spreadsheet Users’ Guide [20]

The resources included are uterotonics for the prevention of PPH (misoprostol or oxytocin), IV fluids, injection set, and extra days in hospital, emergency transport, additional uterotonics, and blood transfusion (Table 4). Bladder catheterisation and repair of tears is not included in the costing of this analysis. The unit price of oxytocin and misoprostol were derived from the International Drug Price Indicator Guide, published by Management Sciences for Health (MSH) (Table 4) [20]. The effective doses used in the analysis are 600 μg misoprostol tablet and ten iU oxytocin administered IV or IM. The costs associated with side effect management, such as shivering and fever, are likely to be small [16] and frequently do not require treatment [21]; so were not included in the economic analysis. Other costs excluded are those that are unlikely to change because of the interventions – staff time, overhead costs and capital costs. Costs were estimated based on 2012 US dollars inflated using the Consumer Price Index (CPI) Inflation Calculator provided by the United States (US) Bureau of Labour Statistics [22].

**Table 4:** Costs used in the model

Costs (2012 US$)	Point estimate	Low	High	Source
Oxytocin/10 iU	$0.2184	0.0368	0.7289	MSH [21]
Misoprostol/200 μg	$0.09	0.0564	0.1786	MSH [21]
IV fluids	$1.79	0.895	3.58	WHO [20]
Infusion-giving set	$0.32	0.16	0.64	WHO [20]
Unit of blood	$41.67	20.835	83.34	WHO [20]
Blood giving set	$0.48	0.24	0.96	WHO [20]
Emergency transport	$0.41/km	0.205	0.82	WHO [20]
Overnight in health centre	$1.39	0.695	2.78	WHO [20]
Overnight in hospital	$3.47	1.735	6.94	WHO [20]
Syringe	$0.07	0.035	0.14	WHO [20]

Costs from the WHO Mother-baby package [20] inflated to 2012 US$ [23]; low values are the point estimates multiplied by 0.5; high values are the point estimates multiplied by 2

The non-drug unit costs are based on the costs in the WHO Mother-Baby Package Costing Spreadsheet from 1999, updated to 2012 US dollars, which may no longer be appropriate. An ideal source of unit costs for the cost consequences analysis would be a comparative in-country study with contemporaneous controls that collects relevant cost data. While such studies are underway, decisions on which strategies to adopt for the prevention of PPH are being taken in countries. In the absence of ideal data, the current study, using inflated unit costs from the WHO Mother Baby Package, provide supportive data that can be used to inform decisions now. In the absence of such estimates, decisions may be opinion or consensus based.

Various one-way (univariate) sensitivity analyses were conducted on key parameters including the incidence of events, cost of drugs and PPH treatment and the proportions of births in the community and hospital settings. The upper and lower confidence limits of the relative risks of treatment from the results of the systematic review Tunçalp [14] were used to substitute the base case treatment effect. The impact of using results from a recent review of oral misoprostol in preventing PPH in the home birth setting was also tested in a sensitivity analysis [23]. Where outcomes were not included in this review (severe PPH, need for blood transfusion), results were generated for the analyses presented here based on the studies included in the review using the random effects model in StatsDirect (Version 2.7.8, 2010) [24–26]. The effects of halving and doubling the treatment costs of PPH on the conclusions were also assessed. The analyses were conducted in Microsoft Excel 2007.

## Results

In the base case estimation (Table 5), it was assumed that 40 % of births would occur in the hospital/health centre setting and 60 % in the community.

**Table 5:** Economic analysis: outcomes in a cohort of 1000 births where oxytocin is and is not available in the hospital setting

Scenario	Oxytocin available in hospital setting			Oxytocin not available		
hospital/community	Oxytocin/Misoprostol	Oxytocin/No treatment	Increment	Misoprostol	No treatment	Increment
Efficacy						
PPH	124	146	−22	123	160	−37
Severe PPH	27	29	−2	27	30	−3
Additional uterotonics	82	88	−6	62	72	−10
Blood transfusion	6	9	−4	2	8	−6
Safety						
Shivering	219	89	130	325	108	217
Fever	56	14	42	86	16	70
Cost						
Drugs for prevention	$249	$87	$162	$270	$0	$270
Treatment of PPH	$2 383	$2 865	-$482	$1 968	$2 770	-$803
Total Cost	$2 633	$2 952	-$320	$2 238	$2 770	-$533

Results are rounded to the nearest whole number. Currency is 2012 US$. Calculations based on 40 % of births occurring in the hospital setting and 60 % in the community setting

Using oxytocin in the hospital setting and misoprostol in the community setting instead of oxytocin in the hospital setting and no treatment in the community setting, 22 cases of PPH could be prevented in the cohort of 1000 women. There would be two fewer cases of severe PPH, six fewer women requiring additional uterotonics and four fewer women would need a blood transfusion. An additional 130 women would experience shivering and 42 women fever. Compared with oxytocin/no treatment, oxytocin/misoprostol was cost saving (US$320; comprising US$162 in extra drug costs, and savings of US$482 associated with treating fewer cases of postpartum haemorrhage).

Similar but more favourable results are obtained if misoprostol is used for all women in the cohort of 1000 births instead of placebo/no treatment in both the hospital and community settings, where oxytocin is not available. Thirty seven cases of PPH could be prevented, there would be three fewer cases of severe PPH, ten fewer women would need additional uterotonics and six fewer women would require a blood transfusion. An additional 217 women would experience shivering and an extra 70 women fever. Using misoprostol in the hospital and community setting would be cost saving (US$533; comprising US$270 in extra drug costs, and US$803 in cost offsets associated with treating fewer cases of PPH).

Table 6 shows the effect of various changes to key parameters used in the economic evaluation for the scenario where oxytocin is used in hospital and misoprostol in community. When using the higher confidence limits of the relative risks for misoprostol versus placebo, only one case of PPH is avoided by using misoprostol in the community and there are additional costs of US$645. Using the relative risks from the recent trials in home birth settings in the Hundley [23] systematic review, the results were more favourable for misoprostol, with more cases of PPH averted (31), fewer side effects (an additional 126 cases of shivering and 33 cases of fever) and greater cost savings ($1477), compared with the base case. Using the higher drug costs in the MSH International Drug Price Indicator Guide [20], misoprostol use in the community is no longer cost saving, and is associated with additional costs of US$151.

**Table 6:** Univariate sensitivity analyses: Incremental outcomes: comparing oxytocin/misoprostol with oxytocin/no treatment in a cohort of 1000 births

Sensitivity analysis	PPH	Severe PPH	Additional uterotonics	Blood transfusion	Shivering	Fever	Total Cost
Base Case	−22	−2	−6	−4	130	42	-$320
Lower bound of RR^a^	−38	−9	−15	−5	109	27	-$792
Higher bound of RR^a^	−1	11	6	0	155	64	$645
Lower cost of drugs	−22	−2	−6	−4	130	42	-$377
Higher cost of drugs	−22	−2	−6	−4	130	42	$151
50 % treatment cost	−22	−2	−6	−4	130	42	-$81
200 % treatment cost	−22	−2	−6	−4	130	42	-$798
Health centre $20; hospital $50/night	−22	−2	−6	−4	130	42	-$1 053
Misoprostol vs placebo RR and placebo baseline risks from Hundley [23]							
Mean	−31	−6	−21	−15	126	33	-$1 477
Lower bound of RR^b^	−45	−7	−27	−16	0	−70	-$1 729
Higher bound of RR^b^	−10	−1	−9	−11	397	918	-$824

^a^Lower and upper 95 % confidence limits for the misoprostol versus placebo relative risks from Tunçalp [14]

^b^Lower and upper 95 % confidence limits for the misoprostol versus placebo relative risks from Hundley [23]

Table 7 summarises the impact of varying the proportion of births that occur in the hospital versus community settings. A higher proportion of births in the community setting resulted in fewer cases of PPH and greater savings associated with misoprostol use, but higher numbers of side effects.

**Table 7:** Sensitivity analyses varying proportion of births in hospital/community: Incremental outcomes (oxytocin/misoprostol minus oxytocin/no treatment) applied to 1000 births

% births in hospital/% births in community	PPH	Severe PPH	Additional uterotonics	Blood transfusion	Shivering	Fever	Total Cost
0/100 %	−37	−3	−10	−6	217	70	-$533
20/80 %	−29	−2	−8	−5	174	56	-$426
40/60 % (base case)	−22	−2	−6	−4	130	42	-$320
60/40 %	−15	−1	−4	−2	87	28	-$213
80/20 %	−7	−1	−2	−1	43	14	-$107
100/0 %	0	0	0	0	0	0	$0

## Discussion

The economic analysis of oxytocin/misoprostol versus oxytocin/no treatment in a mixed clinical setting (40 % hospital/60 % community) demonstrates that a misoprostol prevention strategy can reduce the number of women with PPH, but would result in a large number of women experiencing fever and shivering. The results of the economic analysis are dependent on the proportion of births that occur in hospital compared to the community, as well as drug and PPH treatment costs.

In this study, we incorporated data from the recent Cochrane systematic review [14], international drug prices [20] and the WHO mother-baby costing spreadsheet [19] to provide an economic evaluation applicable in the international context. Although a recent article has questioned the results from misoprostol studies conducted in low- and middle-income countries [12], these studies form the best evidence available and the studies conducted in low-resource settings are more likely to be representative of the use of misoprostol in practice.

The sensitivity analyses show that the price of PPH prophylaxis is important. If the costs of misoprostol and oxytocin are set at the higher end listed by MSH (US$0.1786/200 μg misoprostol and US$0.7289/10 iU oxytocin) [20], there are no longer cost savings associated with misoprostol; there would be an additional cost of US$151 for the prevention of 22 cases of PPH under the hypothetical scenario of 40 % hospital births and 60 % community births. A dose of 600 μg (3 tablets of 200 μg) misoprostol was used in this economic analysis because this is the dosage in the WHO recommendations for the prevention and treatment of postpartum haemorrhage [4] and the clinical data were mainly from 600 μg trials [14, 23]. However, the Tunçalp [14] review suggests that a lower dose of misoprostol (400 μg orally) shows promising results against placebo. Furthermore, as misoprostol side-effects are dose-related [27], use of a lower dose might result in fewer shivering and fever events. The cost-effectiveness of misoprostol is therefore likely to be improved if a lower dose is widely accepted and adopted into clinical practice guidelines.

When using the higher confidence limits of the relative risks for misoprostol versus placebo, 11 more cases of severe PPH were associated with using misoprostol despite a reduction in the number of PPH events (by one). This inconsistent result might be partially explained by the heterogeneity of the trial results. It has been noted [14, 21] that the earlier placebo-controlled misoprostol trials [28–30] did not indicate any reduction in severe PPH compared to placebo, while the more recent trials in Gambia [31], India [24] and Pakistan [32] demonstrated a statistically significant protective effect of misoprostol on severe PPH. These results are consistent with those from the Hundley [23] review in the home birth setting which show a statistically significant reduction in severe PPH for misoprostol compared with placebo. The results of sensitivity analyses using the baseline event rates and relative risk results from Hundley (2013) show a reduction in severe PPH of between one and seven per 1000 women (Table 6).

While there are concerns about the lower effectiveness and safety of misoprostol compared with oxytocin [14], some attention has been given to advance distribution of misoprostol to pregnant women for self-administration following childbirth. It has been argued this strategy has the potential to save lives where no uterotonic coverage exists, but also risks inadvertent use before birth or for other indications, notably abortion [21]. While the comparative benefits and risks of this strategy have not been formally assessed in clinical trials, several observational studies have concluded that community-based education and distribution of misoprostol to pregnant women is safe, acceptable and feasible in low-resource settings [33–35]. Our findings confirm that when oxytocin is available in hospital/health centre settings, misoprostol is not an optimum choice in PPH; while in the community setting, misoprostol could be an effective and cost-saving choice. It needs to be emphasized that misoprostol does not have the same requirements as oxytocin for cold chain storage, sterile equipment, and personnel skilled in parenteral administration. In low-resource settings, a large proportion of births occur in the community setting without professionally trained attendants [8]. Misoprostol may therefore be the only available option in the community setting and other settings where there is a lack of appropriate health resources and no access to the more effective treatment option, oxytocin.

The best information for decision making will come from studies conducted in country, i.e. that take account of the context and specific factors that influence the delivery of care. However such studies are resource intensive and not feasible in many settings. In addition, reliable local cost data may not be readily available for such analyses. The approach used here offers an alternative to aid decision making that takes account of the best available clinical data. The results do not propose a course of action but inform decision makers of the benefits, harms and financial implications of different policy approaches. Sensitivity analyses allow the assessment of the implications of different estimates of effectiveness, harms and costs.

The results of this study need to be interpreted in the light of possible limitations. The first limitation was that for pragmatic purposes, where meta-analysis results were not reported in Tunçalp 2012 due to study heterogeneity, we derived relative risks (and associated 95 % confidence intervals) using the random effects model. Although the random effects model allows for treatment effects to vary across studies, the presence of substantial heterogeneity may mean that it is not appropriate to combine the results of all of these trials. Second, the majority of the resource use and cost data were based on the WHO Mother-Baby Package issued in 1999 [19], which may no longer be appropriate, despite the monetary values being inflated to 2012 US dollars. In the absence of more recent estimates, sensitivity analyses halving and doubling the cost estimates were conducted; the misoprostol scenario remained cost saving (US$81 to US$798). Further, the analysis could be easily adapted to different settings using local resource use and costs. The analysis does not include mortality due to PPH, because of a lack of data, and the quality of life of patients was not considered. Use of misoprostol is associated with uncomfortable side effects (shivering and fever) that are not associated with the use of oxytocin. Alfirevic et al. [36] noted that if used routinely for PPH prevention, misoprostol would be given to very large numbers of women, even though many of them (>90 %) would not experience PPH and therefore not benefit from the drug but be at risk of side effects.

## Conclusions

Oxytocin remains the gold standard treatment for the prevention of PPH but the medicine may not be available and of assured quality in some settings. Based on the best available clinical evidence and economic data, this analysis has demonstrated that use of oral misoprostol in a mixed hospital/community setting can reduce the number of women with PPH and save money. The strategy reduces the numbers of women requiring additional uterotonics and blood transfusions, with cost savings from fewer cases of PPH offsetting the additional costs of misoprostol. These benefits need to be weighed against the large number of additional cases of shivering and fever, although these tend to be mild and self-limiting. The balance of hospital and community births, the cost of misoprostol and the costs of treating PPH will influence the economic decision in different countries.

## Abbreviations

AMTSL: Active management of the third stage of labour
IM: Intramuscular
iU: international units
IV: Intravenous
MSH: Management Sciences for Health
PPH: Postpartum haemorrhage
US: United States
WHO: World Health Organization
